# Biophysically based computational models of astrocyte ~ neuron coupling and their functional significance

**DOI:** 10.3389/fncom.2013.00044

**Published:** 2013-05-07

**Authors:** John Wade, Liam McDaid, Jim Harkin, Vincenzo Crunelli, Scott Kelso

**Affiliations:** ^1^Intelligent Systems Research Centre, University of UlsterDerry, UK; ^2^Cardiff School of Biosciences, Cardiff UniversityCardiff, UK; ^3^Center for Complex Systems and Brain Sciences, Florida Atlantic UniversityBoca Raton, FL, USA

Neuroscientists are becoming increasingly interested in modeling brain functions however, capturing underlying biophysical mechanisms requires plausible, biologically realistic models at the cellular level. Moreover, the (often conflicting) demands of biological realism and computational tractability have to be accommodated. Recent publications highlight the interaction of astrocytes with multiple synapses: if we are to fully appreciate this dynamic and coordinated interplay between cells, then more research on bidirectional communication between astrocytes and neurons is required. For example, a better understanding of astrocyte-neuron coupling may lead to providing building blocks for studying the regulatory capability of astrocytic networks on a larger scale. This special topic presents nine papers which cover a range of issues from computational models of astrocyte-neuron interactions to the role of astrocytes in neurological disorders.

In the paper by Gordleeva et al. (2012) the basic physiological functions of tripartite synapses and astrocytic regulation at the level of neural network activity is considered. The paper concludes that astrocytes support homeostatic regulation of network activity by modulating neural networks into a bistable regime of activity with two stable firing levels and spontaneous transitions between them. The comprehensive review article by Volman et al. (2012) deals with the premise that pathways for astrocyte-neuron interaction can enhance the information processing capabilities of brains, yet in other circumstances may lead the brain on the road to pathological ruin. The paper focuses on existing computational models of astrocytic involvement in epileptogenesis and their relevance to existing physiological data. Similarily, Min et al. (2012) review recent experimental literature on astrocyte-neuron interactions and discuss the dynamic effects of astrocytes on neuronal excitability and short- and long-term synaptic plasticity. Their paper also addresses potential computational functions of astrocyte-neuron interactions in the brain, in particular how astrocytes may enhance the computational power of neuronal networks in previously unexpected ways. In the paper by Allam et al. (2012) a computational model is extended to show that glial transporters modulate synaptic transmission mediated by ionotropic AMPA and NMDA receptors at glutamatergic synapses. Their results demonstrate that astrocytes may play a crucial role in spike timing dependent processes with important implications for neurological diseases. Le Meur et al. (2012) use whole-cell recordings in rat acute brain slices and electron microscopy to test whether hippocampal astrocytes release the inhibitory transmitter GABA. They observe that slow transient inhibitory currents share characteristics with the slow NMDA receptor-mediated currents shown previously to result from astrocytic glutamate release. Their results provide quantitative characteristics of astrocyte-to-neuron GABAergic signaling and suggest that all principal neurons in the hippocampal network are under a dual, excitatory and inhibitory, influence of astrocytes. The relevance of astrocytic release of GABA and glutamate on the pathophysiology of the hippocampus remains to be established. The paper by De Pittà et al. (2012) considers the plausibility of astrocytes performing information processing by calcium signaling and hence their role in setting the basal tone of synaptic transmission. The work presents a theoretical treatment focusing on the modulation of synaptic release probability by the astrocyte and its implications for synaptic plasticity. The analysis of signaling pathways underlying such modulation refines the tripartite synapse model and has profound implications on our understanding of brain function.

In the paper by Wade et al. (2012) it is shown that retrograde signaling via astrocytes may underpin self-repair in the brain. Their model of self-repair is based on recent research showing that retrograde signaling mediated by astrocytes increases the probability of neurotransmitter release at damaged or low transmission probability synapses. Model simulations demonstrate that astrocytes are capable of bidirectional communication with neurons and that indirect signaling through retrograde messengers, such as endocannabinoids, leads to modulation of synaptic transmission probability. This opens up the possibility of developing highly adaptive, distributed computing systems that can, at fine levels of granularity, fault detect, diagnose, and self-repair autonomously. Reato et al. (2012) present a study of a parsimonious network model of neurons and astrocytes consisting of excitatory and inhibitory neurons described by Izhikevich-like neuron dynamics. Experimentally observed Ca^2+^ change in astrocytes in response to neuronal activity is modeled using linear equations. Their paper suggests that glutamate release from astrocytes above certain intracellular Ca^2+^ concentrations provides a non-linear positive feedback signal to neurons. The threshold of focal ictal discharge (ID) generation is lowered when an excitatory feedback-loop between astrocytes and neurons is included. Simulations show that astrocytes can contribute to ID generation by directly affecting the excitatory/inhibitory balance of the neuronal network. Witcher and Ellis (2012) highlights the fact that more than one-third of all epilepsy patients have incompletely controlled seizures or debilitating medication side effects in spite of optimal medical management. The cellular mechanisms underlying seizure activity are not completely understood. Their paper describes multiple lines of evidence supporting the likely contribution of astroglia to epilepsy, focusing on individual astrocytes and their network functions. Witcher and Ellis (2012) conclude that astrocytes are likely important targets for the developing field of neuromodulation in the treatment of epilepsy.

In short, while the papers in this special topic are representative of current thinking in the field, they also draw attention to the fact that research into astrocytes and their interaction with neurons is still very much in the embryo stage.
